# Enhanced Antitumor Efficacy of a Vascular Disrupting Agent Combined with an Antiangiogenic in a Rat Liver Tumor Model Evaluated by Multiparametric MRI

**DOI:** 10.1371/journal.pone.0041140

**Published:** 2012-07-18

**Authors:** Feng Chen, Yingmei Feng, Kaier Zheng, Frederik De Keyzer, Junjie Li, Yuanbo Feng, Marlein Miranda Cona, Huaijun Wang, Yansheng Jiang, Jie Yu, Guy Marchal, Catherine Verfaillie, Bart De Geest, Raymond Oyen, Yicheng Ni

**Affiliations:** 1 Theragnostic Laboratory, Department of Imaging and Pathology, University Hospital, University of Leuven, Leuven, Belgium; 2 Department of Radiology, Zhong Da Hospital, Southeast University, Nanjing, China; 3 Interdepartmental Stem Cell Institute, University of Leuven, Campus Gasthuisberg, Leuven, Belgium; 4 Department of Radiology, BenQ Medical Center, Nanjing Medical University, Nanjing, China; 5 Center for Molecular and Vascular Biology, University of Leuven, Campus Gasthuisberg, Leuven, Belgium; National Institutes of Health, United States of America

## Abstract

A key problem in solid tumor therapy is tumor regrowth from a residual viable rim after treatment with a vascular disrupting agent (VDA). As a potential solution, we studied a combined treatment of a VDA and antiangiogenic. This study was approved by the institutional ethical committee for the use and care of laboratory animals. Rats with implanted liver tumors were randomized into four treatment groups: 1) Zd6126 (Zd); 2) Thalidomide (Tha); 3) Zd in combination with Tha (ZdTha); and 4) controls. Multiparametric MRIs were performed and quantified before and after treatment. Circulating endothelial progenitor cells (EPCs) and plasma stromal cell-derived factor-1α (SDF-1α) were monitored. Tumor apoptosis, necrosis, and microvessels were verified by histopathology. A single use of Zd or Tha did not significantly delay tumor growth. The combined ZdTha showed enhanced antitumor efficacy due to synergistic effects; it induced a cumulative tumor apoptosis or necrosis, which resulted in significant delay in tumor growth and reduction in the viable tumor rim; it also reduced tumor vessel permeability; and it improved tumor hemodynamic indexes, most likely via a transient normalization of tumor vasculature induced by Tha. A stepwise linear regression analysis showed that the apparent diffusion coefficient was an independent predictor of tumor growth. We found no significant increases in Zd-induced circulating EPCs or plasma SDF-1α. ZdTha showed improved therapeutic efficacy in solid tumors compared to either agent alone. The therapeutic effects were successfully tracked *in vivo* with multiparametric MRI.

## Introduction

Tumor vasculature has become an attractive target for therapy, because blood vessels are crucial for maintaining tumor growth with a supply of oxygen and other nutrients, and provides a primary escape route for metastases. Antivascular approaches with vascular disrupting agents (VDAs) have recently emerged as a novel antitumor strategy [1], [2]. VDAs aim to cause rapid, selective shutdown of existing tumor vessels by selectively disrupting the microtubules of the cytoskeleton in endothelial cells; this leads to ischemic necrosis of the central cells of the tumor [3]. Several small molecular VDAs, including CA-4-P and Zd6126, are currently in clinical trials or undergoing preclinical testing [4]. However, tumors can rapidly regrow from the residual viable rim when VDAs are used alone; this compromises the therapeutic utility of these agents [2].

The effect of VDAs might be improved by combining them with other approaches to prevent tumor rebound, like conventional chemotherapies, radiotherapies, or recently described antiangiogenic agents [5]. In practice, treatments with antiangiogenics alone have been found insufficient, because they impair only one aspect of tumor angiogenesis. Therefore, current efforts have gradually shifted from single use to combinations of agents in both preclinical and clinical settings [6], [7]. In theory, the combination of VDAs and antiangiogenics holds great promise, because VDAs will induce acute vascular collapse, and antiangiogenics inhibit recruitment and growth of new tumor vessels. Thus, the two approaches are likely to have synergistic therapeutic efficacy.

Advances in the development of new anti-tumor strategies have highlighted the need for *in vivo* evaluation of tumor response with imaging biomarkers. For example, magnetic resonance imaging (MRI), an established non-invasive technique for monitoring tumor treatment response, offers a wide selection of parameters that serve as biomarkers. MRI biomarkers include tumor size, therapy-induced necrosis, and hemodynamic changes observed in the evaluation of tumor treatments [8]. Currently, this type of multiparametric tumor imaging is an emerging paradigm in evaluating tumor responses to therapy [9].

In the present study, we tested a new antitumor strategy by combining a VDA, Zd6126 (Zd), and an antiangiogenic agent, Thalidomide (Tha). Zd is a phosphate prodrug of the tubulin-binding agent, Zd phenol, a small molecular inhibitor of microtubule polymerization [10]. Tha is not a classic vascular endothelial growth factor-targeting agent, but was recently demonstrated to have antiangiogenic and immunomodulating properties [11], [12]. Thus, Tha has become a common antiangiogenic therapy in both preclinical and clinical settings [13], [14]. We hypothesized that the complementary effects of these agents would strongly inhibit, or even prevent, tumor regrowth. To our knowledge, the combination of VDA and Tha has not been tested for treating solid tumors. Here, we tracked and evaluated the therapeutic effects of this combination in a rat liver tumor model with a published multiparametric MRI protocol [15].

## Materials and Methods

### Animal Model

This study was approved by the institutional ethical committee for the use and care of laboratory animals. We used adult WAG/Rij rats (Iffa Credo, Brussels, Belgium) with existing subcutaneous rhabdomyosarcomas as donors. The tumor tissues were excised and implanted into a tumor-naive set of rats in our laboratory. We performed liver tumor implantations in 48 tumor-naive WAG/Rij rats that weighed 225 g to 275 g, as described previously [16], which mimics a hypervascular human liver metastasis.

### Experimental Design

**Figure 1:**
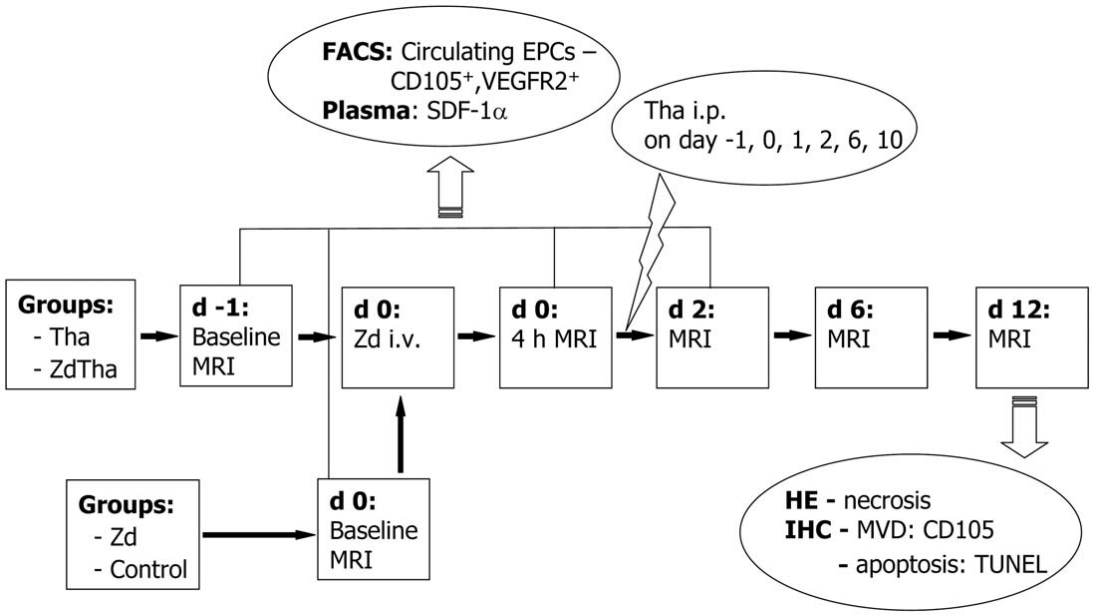
Study design. Rats were randomly assigned into the following 4 groups: (group 1) Zd alone (n = 11); (group 2) Tha alone (n = 11); (group 3) ZdTha (n = 12); (group 4) untreated controls (n = 10). For groups 2 and 3, Tha (200 mg/kg) was injected intraperitoneally (i.p.) six times, on days (d) -1, 0, 1, 2, 6, and 10. For groups 1 and 3, Zd (50 mg/kg) was injected once intravenously (i.v.) on day 0. The controls were injected on days 0, 1, 2, 6, and 10 with the vehicles (solvents) of both agents (i.v. and i.p.) at the same time points that the other groups were injected. For all groups, MRI was performed before, and at 4 h, 2 d, 6 d, and 12 d after the initial treatment. Before the baseline, 4 h, and 2 d MRIs, rat tails were incised to collect blood samples for monitoring the levels of circulating EPCs and plasma SDF-1α in both Zd and ZdTha groups. At the end of the experiment, animals were sacrificed for histopathology examinations. (Zd, Zd6126; Tha, Thalidomide; ZdTha, Zd6126+Thalidomide; i.p., intra-peritoneal injection; i.v., intravenous injection; MRI, Magnetic resonance imaging; FACS, Fluorescence-activated cell sorting; EPCs, endothelial progenitor cells; VEGFR, vascular endothelial growth factor receptor; SDF-1α, stromal cell-derived factor-1α; HE, hematoxylin-eosin staining; IHC, Immunohistochemistry; MVD, microvessel density; TUNEL, terminal deoxynucleotidyl transferase biotin dUTP nick end labeling).

The rats were randomly assigned into the following 4 groups (n = 12 in each group; Fig.1): 1) *Zd group*: The VDA, Zd (AstraZeneca, Cheshire, UK), was dissolved with 4 portions of 8.4% sodium carbonate and 1 portion of phosphate-buffered saline (PBS), pH 7.4. On day 0, one dose of 50 mg/kg Zd was injected intravenously (i.v.) into each animal; 2) *Tha group*: Stock solutions of the antiangiogenic agent, Tha (Pharmaceutical Factory, Changzhou, China), were prepared in DMSO (Sigma-Aldrich NV/SA, Bornem, Belgium). The stock solution was injected intraperitoneally (i.p.) at a dose of 200 mg/kg, six times at regular intervals during the experiment; [17] 3) *ZdTha group*: first dose of Tha was injected 24 h in advance of Zd; 4) *control group*: animals were i.v. and i.p. injected with the vehicles (solvents) of both agents at the same time points that the other groups were injected. For all groups, MRI was performed before, and at 4 h, 2 days (d), 6 d, and 12 d after the initial treatment. Before the baseline, 4 h and 2 d MRI, rat tails were incised to collect blood samples for monitoring the levels of circulating endothelial progenitor cells (EPCs) and plasma stromal cell-derived actor-1α (SDF-1α) in both Zd and ZdTha groups. This is to find if there is any increased circulating EPCs induced by a VDA. [18] At the end of the experiment, animals were sacrificed for histopathology examinations.

### MRI Setup

Optimal image quality in small animals with a large-bore human MRI magnet requires a number of optimizations. First, all imaged animals must be as close as possible to the center of the magnet, both along the axis of the magnet and in the transverse plane. We constructed a supportive structure to allow supine positioning of the animals in the exact center of the magnet, with a tube attachment to the gas anesthesia machine outside the MR room.

MRI scans were performed with a clinical 1.5T system (Sonata, Siemens, Erlangen, Germany) with a maximum gradient capability of 40 mT/m. All imaging was performed with a commercially available, four-channel, phased-array, wrist coil (MRI Devices Corporation, Waukesha, WI, USA). The coil fit close to the animal, and allowed acquisition of high signal images, adequate for anatomical imaging. It also allowed the use of parallel imaging, which was performed to shorten the imaging time, reduce artifacts from breathing and movements, and improve signal homogeneity.

The rats were anesthetized with inhalation of 2% isoflurane. For all rats, the following sequences were acquired in the transverse plane, with a slice thickness of 2 mm and an inter-slice gap of 0.2 mm in the following order:

1. T2-weighted fast spin echo imaging (T2WI) with fat saturation and a repetition/echo time (TR/TE) of 3860/106 ms, a turbo factor of 19, a field of view (FOV) of 140 × 70 mm, and an acquisition matrix of 256 × 256. Three signals were acquired, in a scan time of 1 min 25 sec.

2. Diffusion weighted imaging (DWI) with a 2-dimensional (2D), spin echo, echo-planar imaging sequence. We used a TR/TE of 1700/83 ms, a FOV of 140 × 82 mm, and an acquisition matrix of 192 × 91 (in-plane resolution: 0.7 × 0.9 mm). For the DWI, six signals were acquired, including repeated measurements for 10 different *b* values (0, 50, 100, 150, 200, 250, 300, 500, 750, and 1000 s/mm^2^) in three directions (x, y, and z) and averaged for the calculation of the isotropic apparent diffusion coefficient (ADC) value. A parallel imaging technique was applied to reduce susceptibility artifacts and examination times. The total examination time was 4 min 51 s.

3. Dynamic contrast enhanced MRI (DCE-MRI) using a fat saturated 3D T1-weighted gradient echo sequence (volumetric interpolated breath-hold examination, VIBE). The following parameters were used: a TR/TE of 7.02/2.69 ms, a FOV of 81.3 × 130 mm, parallel imaging with an acceleration factor of two and a matrix of 154 × 192 (in-plane resolution: 0.5 × 0.7 mm). In total, 80 measurements were acquired, each lasting 3.7 sec, leading to a total scan time of 4 min 58 sec. A bolus of 0.04 mmol/kg gadoterate meglumine (Dotarem®, Guerbet, France), prepared with a gadolinium concentration of 0.5 mmol/ml was injected i.v. after the 20^th^ measurement.

4. Dynamic susceptibility contrast MRI (DSC-MRI) with a T2*- weighted echo-planar imaging sequence with the following parameters: a TR/TE of 2000/46 ms, a FOV of 140×70 mm, parallel imaging with an acceleration factor of two and a matrix of 128×128; (in-plane resolution: 1.1×0.5 mm). In total, 80 measurements were acquired, each lasting 2 sec, leading to a scan time of 2 min 46 sec. An i.v. bolus of 0.3 mmol/kg Dotarem ® was given after the 20^th^ measurement.

5. Contrast-enhanced fat saturated T1-weighted fast spin echo imaging (CE-T1WI) immediately after the DSC-MRI sequence, with the following parameters: a TR/TE of 535/9.2 ms, a turbo factor of seven, a FOV of 140 × 70 mm, and an acquisition matrix of 256 × 256 (in-plane resolution: 0.5 × 0.3 mm). Four signals were acquired, in a scan time of 1 min 24 sec.

The total examination time for the whole MRI protocol was 15 min 35 s.

### Fluorescence-activated Cell Sorting (FACS) and Enzyme-linked Immunosorbent Assay (ELISA)

FACS was used to detect circulating endothelial progenitor cells (EPCs) in the blood. Mononuclear cells were isolated from 100 µl of peripheral blood with Lymphoprep™ (Nycomed Pharma, Roskilde, Denmark). After washing with PBS, cells were stained with rabbit anti-mouse Flk-1 (1 µg/10^6^ cells, sc-315, Santa Cruz Biotechnology, Santa Cruz, US) and goat anti-mouse CD105 (2.5 µg/10^6^ cells, AF1320, R&D, Minneapolis, US) at 4°C for 30 min. This was followed by the addition of donkey anti-rabbit Alexa 488 (1/400, Invitrogen, Grand Island, US) and donkey anti-goat Alexa 568 (1/400, Invitrogen, Grand Island, US) and incubation for 20 min before FACS analysis (FACS Calibur, BD, San Jose, US). EPCs were identified as CD105^+^ Flk-1^+^ cells and quantified with CellQuest software (BD, San Jose, US). For the control, we used isotype-matched control antibodies (BD, San Jose, US) [19].

The plasma level of SDF-1α was determined with an ELISA kit (R&D, Minneapolis, US), according to the manufacturer’s instructions.

### Tissue Processing, Immunohistochemical (IHC) Staining, and Histology

At the end of the experiment (day 12), all rats were sacrificed for tissue processing, immunohistochemical staining, and histology. First, animals were deeply anesthetized with an intraperitoneal injection of pentobarbital (50 mg/kg) (Nembutal, Sanofi Sante Animale, Brussels, Belgium). Then, animals were transcardially perfused through a ventricular catheter with saline, followed by 4% paraformaldehyde (PFA) under gravity flow for 10 min. The livers were collected, fixed with formalin, embedded in paraffin, and sliced into transverse sections. The sections were 2 mm thick, and were positioned on the same planes used for the MRI scans, based on a grid (Agar Scientific, England). The tumor slices (5 µm thick) were stained with hematoxylin and eosin (HE). Other sections were stained with goat anti-mouse CD105 (10 µg/ml, AF1320, R&D, US) overnight and then amplified with Tyramide signal amplification (Perkin Elmer) to detect neoangiogenesis. In parallel, isotype control of CD105 was performed by omitting primary antibody. To study apoptosis, sections were stained with terminal deoxynucleotidyl transferase biotin dUTP nick end labeling, (TUNEL) from an apoptosis detection kit (KeyGen Biotech, Nanjing, China).

### MRI Analysis

*(see Supporting Information, Text S1)*.

Image analyses were performed off-line on a LINUX workstation with dedicated software (Biomap, Novartis, Basel, Switzerland). To obtain robust measurements and to facilitate comparisons between different treatment approaches, we opted to measure the entire tumor, including the viable rim and necrotic areas. Free-hand regions of interest (ROI) were used to delineate the entire tumor on MR images. A round ROI was used to define a region of normal liver tissue for the purpose of comparison with tumor. All ROIs were larger than 10 pixels in size. The delineation was performed by two experienced radiologists in consensus. For each morphological and functional MR parameter, tumor and normal liver were measured with ROIs on all tumor-containing image slices, and mean values were obtained for each tumor and liver respectively. After that, the average and standard deviation for each parameter were calculated for each group at each time point for statistical analysis.

#### Tumor volume

For each lesion, the tumoral areas were delineated on T2WI with operator-defined ROI on all slices and automatically combined into the total tumor volume. The tumor volume change (%) was calculated with the following formula: [(volume_post_ - volume_pre_)_/_volume_pre_] ×100.

#### Tumor viable rim and necrosis

The residual viable tumor rim after treatment was visualized as a contrast-enhanced, high signal region on the CE-T1WI. The relative viable rim (100× maximum rim diameter/maximum tumor diameter; expressed in %) was calculated for each tumor only from 2 days after treatment. In both Tha and control groups, direct measurements of tumor viable rims were not available, due to the inhomogeneous, irregular distribution of necrosis. Instead, the tumor necrosis area was contoured on CE-T1WIs, based on the unenhanced, low-signal area within the tumor that was observed after injection of a contrast agent. Relative volumes (%) of tumor necrosis were calculated by normalizing them to the entire tumor volume.

Pixel-wise ADC (mm^2^/sec) calculations were automatically performed by the built-in software of the scanner according to a mono-exponential ADC calculation model (Text S1) using all 10 b values [20]. Similar to the tumor volumetry, each tumor was completely delineated on all slices of these ADC maps to calculate a whole tumor ADC; this was then normalized to the ADC of healthy liver tissue to generate a relative ADC (rADC). Then, rADC changes (%) were calculated as [(rADC_post_ - rADC_ pre_)_/_rADC_pre_] ×100.

### Functional Parameters Derived from the DCE-MRI

We derived functional parameters from the DCE-MRI, including the volume transfer constant (K^trans^, min^-1^) and the fractional volume of the extravascular space (v_e_, %). These parameters were obtained with a standard method based on the Tofts model [21] (Text S1).

### Parameters Derived from the DSC-MRI

A kinetic analysis of the contrast agent distribution in DSC-MRI is typically based on the tracer-dilution theory [22]. Functional parameters were derived from the DSC-MRI, including relative blood volume (rBV, arbitrary unit) and relative blood flow (rBF, arbitrary unit). These can be estimated by using a previously reported deconvolution method [23] (Text S1).

### Microscopic Analysis

Microscopic image analyses were performed blinded to the experimental details. On IHC and HE stained sections, image analysis software (ImageJ 1.34 s, NIH, US) was used to quantify the percentages of TUNEL positive brown stained apoptosis and amorphous eosinophilic necrosis in the total tumor area. To quantify neoangiogenesis, tumor sections were visualized with an Axiovert 200 M microscope (Carl Zeiss Inc, Gottingen, Germany) and captured with a Zeiss Axiocam digital camera connected to the microscope (AxioVision 4.7 software). The number of fields analyzed per tumor sample ranged from 3 to 10, depending on tumor size; the fields analyzed were representative of all microvessel densities (MVD) [24]. Briefly, whole tumor sections were scanned at a magnification of 50× with a MoSAIC acquisition technique (AxioVision 4.7) to identify areas with microvessels. Then, the number of CD105-fluorescent, immunopositive microvessels per field were counted with a fluorescence microscope at a power of 400× with computer assisted image analysis and KS300 software. The MVD was calculated as the number of microvessels per square micron. The final MVD for each tumor was expressed as the mean MVD value over all examined fields.

### Statistical Analysis

Statistical analysis was carried out with the SPSS for windows software package (release 18.0, SPSS Inc., Chicago, US). A general linear model, with repeated-measures, was used to compare changes in various parameters over time among groups. The nonparametric Kruskal-Wallis analysis of variance was performed for comparing parameters between groups at certain time points, followed by post-hoc group-wise comparisons using a Bonferroni correction for multiple testing. A stepwise multivariate linear regression analysis was performed to identify independent predictors of tumor volume change. All data were presented as mean ± standard deviation and a *P* value less than 0.05 was considered statistically significant.

## Results

### General Aspects

A total of 48 rats were subjected to liver tumor implantation, but 4 rats died prior to randomization due to the anesthesia. A total of 44 rats were randomly assigned to the study groups, as follows: 10 rats in the control group; 11 in the Zd group; 11 in the Tha group; and 12 in the ZdTha group.

Eight rats (4 in the Tha group, 4 in the ZdTha group) were found to have minor hemorrhaging around the eye socket and perianal area at 1 d after the first Tha treatment. This was probably due to a venous thromboembolism induced by Tha [25].

### ZdTha Induced the Largest Reductions in Tumor Volume Growth

**Figure 2:**
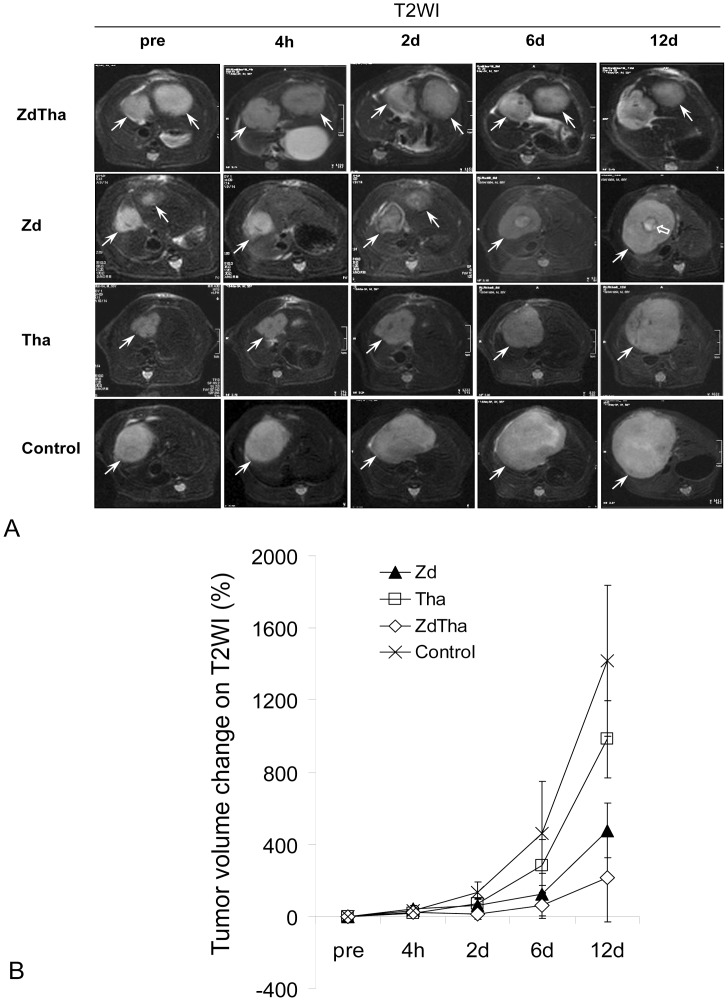
Tumor growth delay after treatments. Among the four treatment groups, ZdTha induced the longest delay in tumor growth over the examined period. (**A**) Representative axial images of liver tumors, acquired with T2-weighted images (T2WI) (TR/TE = 3860/106 ms). **Top row:** The tumors (arrows) in both left and right liver lobes showed very little growth over the examined period of ZdTha treatment. **Row 2:** The right tumor regrew rapidly from 2 d after Zd treatment; note the reduction in central necrosis (open arrow); **Row 3:** The tumor grew remarkably during Tha treatment; **Bottom** **row:** The tumor grew significantly in the control group. (**B**) Tumor volume changes after treatment compared to pretreatment measured on T2WI in the four treatment groups (mean ± standard deviation). Between 2 d and 12 d after treatment, the ZdTha group exhibited significantly smaller tumor volume changes compared to the other 3 groups (*P*<0.05) (Table S1).

As shown on T2WI tumor volumetry, Tha only had an early and brief effect on the tumor growth (*P = *0.0007 at 2 d, and *P*>0.05 at all other time points), but Zd and ZdTha both induced a significant and persisting smaller tumor volume from 2 d to 12 d after administration, compared to the controls (*P*<0.001 for both). Furthermore, ZdTha performed significantly better than Zd in delaying tumor growth from 2 d to 6 d after treatment (*P*<0.05). However, at 12 d after treatment, there was no longer a significant difference (*P*>0.05) in tumor volume changes between the Zd and ZdTha groups, due to the regrowth of tumors (Fig. 2, Table S1).

### ZdTha most Effectively Induced Tumor Apoptosis and Necrosis, and Reduced Viable Rim Size

#### CE-T1WIs

The mean relative viable rim in the ZdTha group was significantly smaller than that in the Zd group at 6 d and 12 d after treatment (*P = *0.0427 and *P = *0.0050, respectively). Accordingly, the mean relative tumor necrosis volume visualized on CE-T1WIs in the ZdTha group was significantly larger compared to that of the Zd group at 6 d and 12 d after treatment (*P* = 0.0026 and *P* = 0.0140, respectively) (Fig. S1).

#### ADC maps

At 4 h: Compared to controls, both the ZdTha and Zd groups showed a significant drop in the rADC (*P*<0.0001 for both), due to the vascular shutdown induced by Zd. No significant difference of rADC was seen between the Tha group and controls (*P* = 0.181).

At 2 d: The Zd-induced tumor necrosis caused a rise in the rADCs of both ZdTha and Zd groups. However, the increases were significantly greater than the rADCs of controls only in the ZdTha group (*P = *0.0003), not in the Zd group (*P = *0.0751). In addition, the rADC was much higher in the ZdTha than in the Zd group (*P = *0.0256). In contrast, the rADC in the Tha group was significantly reduced compared to controls (*P* = 0.0003).

At 6 d: Only the rADC of the ZdTha group was significantly higher (*P* = 0.0386) than that of the controls; the rADCs in the Zd and Tha groups were not significantly different compared to controls (*P* = 0.1229, *P* = 0.0858, respectively). However, a sharp increase in rADC was observed in the Tha group compared to that observed at 2 d.

**Figure 3:**
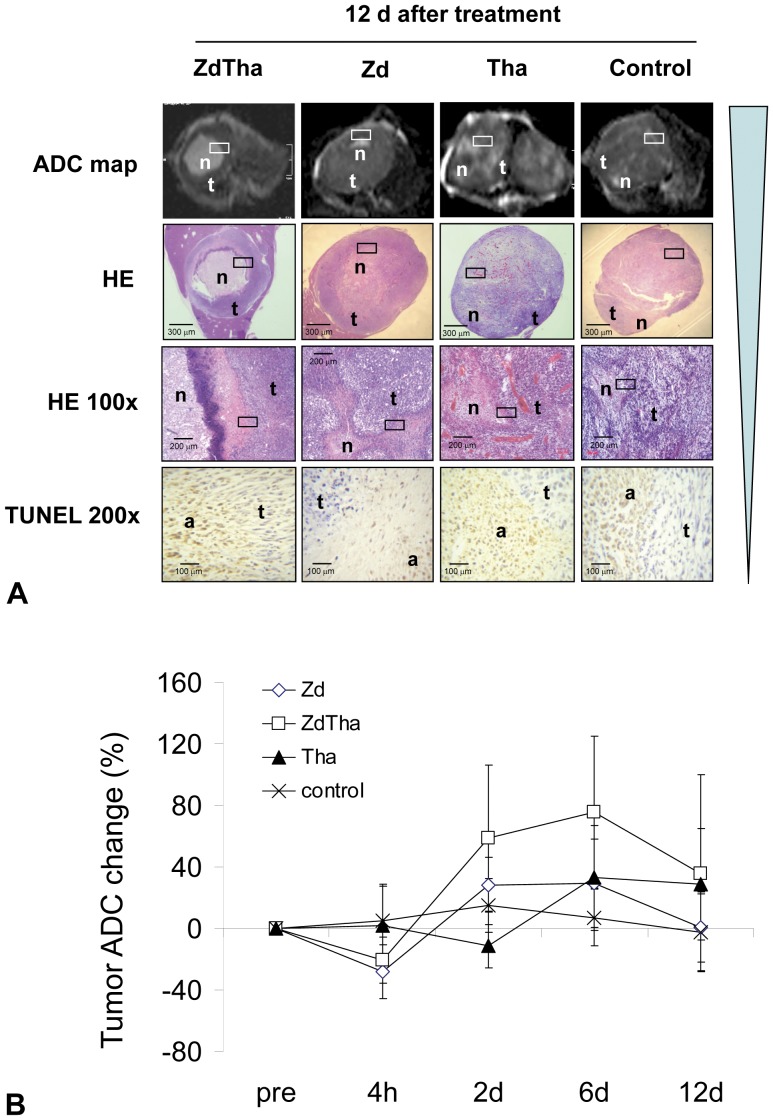
ZdTha increased the tumor ADC value, apoptosis, and necrosis. (**A**) Representative axial ADC maps (calculated from DWI (TR/TE = 1700/83 msec) with 10 b values between 0 and 1000 sec/mm^2^) of liver tumors at 12 d after treatment. **Top row**: significant necrosis (n) was apparent within the tumor (t) in ZdTha and Tha groups compared to Zd and control groups; **Row 2:** A hyperintense area on the ADC map in top row was verified as necrosis (n) on macroscopic HE stained slices. The viable tumor (t) was consistent with that seen in ADC maps. **Row 3:** High magnification microscope (HE 100×) further confirmed the findings of necrosis (n) and viable tumor (t) seen on ADC maps and macroscopic HE slices. **Bottom row:** The apoptosis (a) and viable tumor cells (t) on high magnification (TUNEL 200×) were consistent with the findings of necrosis (n) and viable tumor (t) seen on high magnification HE slices (100×). Rectangular frames indicate the areas shown at high magnification (HE 100×, TUNEL 200×). (**B**) Dynamic changes in the ADC values occurred over the entire experiment in all four groups. Among four groups, only the change of tumor ADC in ZdTha group was significantly higher than that of controls from 2 d to 6 d after treatment, indicating more necrosis was induced by combined ZdTha therapy (Table S2).

At 12 d: Due to the regrowth of tumors, the rADCs in the ZdTha and Zd groups began to decrease from 6 d to 12 d. However, the rADC remained unchanged in the Tha group. Consequently, the rADCs in the ZdTha and Tha groups were much higher than those observed in both the Zd group (*P = *0.0251, *P = *0.0026, respectively) and the control group (*P = *0.0190, *P = *0.0160, respectively) (Fig. 3, Table S2).

#### HE staining

**Figure 4:**
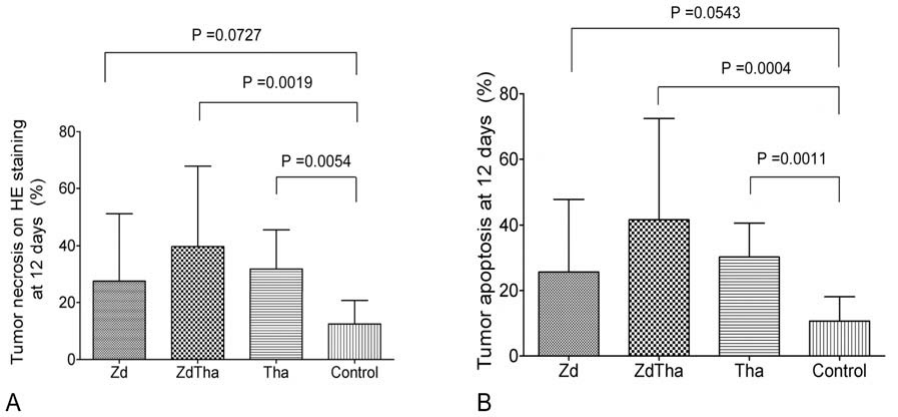
Tumor necrosis and apoptosis at 12 d after treatment. (**A**) The percentages of necrotic compared to total tumor areas detected on HE-stained tumor sections were significantly higher in ZdTha and Tha groups compared to the control group (*P = *0.0019 and *P = *0.0054, respectively). (**B**) Similarly, the percentages of apoptotic areas compared to total tumor areas detected on immuno-stained tumor sections were significantly higher in ZdTha and Tha groups compared to the control group (*P = *0.0004 and *P = *0.0011, respectively).

Twelve days after treatment, the percentages of necrotic compared to total tumor areas on HE stained tumor sections were significantly higher in both ZdTha and Tha groups compared to the control group (*P = *0.0019 and *P = *0.0054, respectively). In contrast, no significant difference was found in the necrotic areas of the Zd and control groups (*P = *0.0727) (Fig. 4A).

#### Apoptosis on IHC stained slices

Similar to findings with HE staining, the percentages of apoptotic areas compared to total tumor areas on IHC stained tumor sections were significantly higher in both ZdTha and Tha groups compared to the control group (*P = *0.0004 and *P = *0.0011, respectively). In contrast, no significant difference was found in the apoptotic areas of the Zd and control groups (*P = *0.0543) at 12 d after treatment (Fig. 4B).

### ZdTha Prolonged the Reduction of Tumor Hemodynamic Indexes

**Figure 5:**
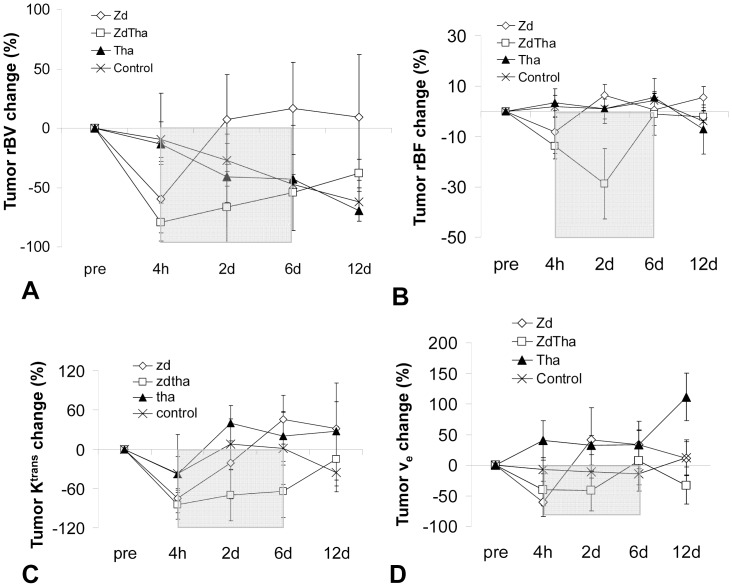
Changes of tumor hemodynamic indexes after treatments. ZdTha inhibited tumor growth with prolonged reductions of tumor relative blood volume (rBV), relative blood flow (rBF), and tumor vessel permeability. (**A**) The rBV change in the ZdTha group remained at a lower level than that of the Zd group (*P = *0.0003) until day 6, then it increased at 12 d. (**B**) A significant reduction in tumor rBF at 2 d was noted in the ZdTha group, but not the other 3 groups (*P*<0.05 for all 3 groups). (**C**) The tumor volume transfer constant (K^trans^) change was much lower In the ZdTha group compared to controls at 4 h (*P*<0.0001), 2 d (*P = *0.0026), and 6 d (*P* = 0.0500). This indicated that combined ZdTha therapy enhanced the transient reduction in tumor vessel permeability. (**D**) Compared to controls, the ZdTha group maintained low tumor fractional volumes, v_e,_ from 2 d (*P = *0.0420) to 12 d (*P = *0.0812), except at 6 d. In the Tha group, v_e_ sharply rose at 12 d. This group exhibited a relatively large amount of necrosis, which may cause an increase in extracellular fluid, compared to the other groups. The rectangular shadow indicates the transient window of tumor vessel normalization induced by the combined ZdTha treatment (Tables S3, S4).

**Figure 6:**
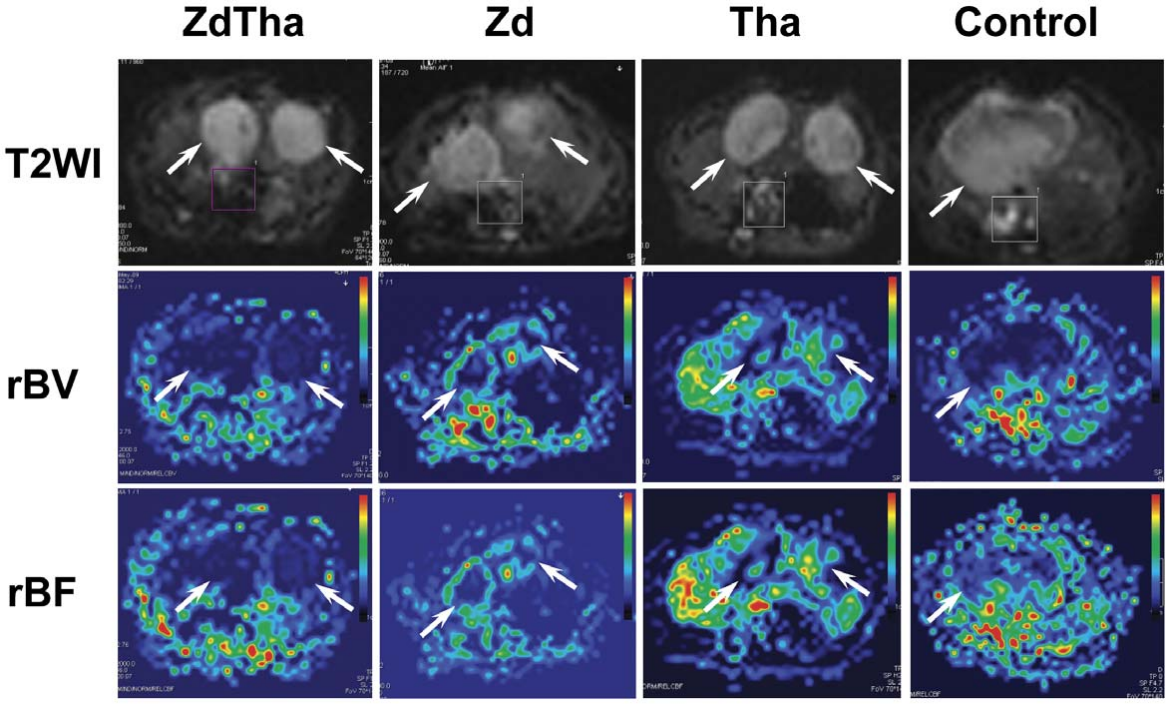
Representitive rBV and rBF maps at day 2 after treatment. **Top row:** T2W images for anatomical visualization of tumors (arrow). **Middle row:** the same slice as shown at T2W images, rBV maps showed reduced level of rBV at various extents in tumors (arrow) from four groups. **Bottom row:** the same slice as shown above, rBF maps showed reduced level of rBF at various extents in tumors (arrow) from four groups.

Compared to the controls, tumor rBV decreased dramatically at 4 h, presumably due to a rapid vascular shutdown induced by Zd in both the Zd and ZdTha groups (both *P*<0.0001). This was followed by a rapid rebound at 2 d in the Zd group (no longer significantly different compared to controls; *P* = 0.4003), but not in the ZdTha group (significantly lower than controls; *P = *0.0020). The rBVs in the ZdTha group remained at a lower level than those of the Zd group (*P = *0.0003) until day 6, but this difference become non-significant at 12 d (*P* = 0.0979). In contrast, both Tha and control groups showed comparable decreases in the rBV over time (Fig. 5A, Fig.6, Table S3). A significant reduction in tumor rBF at 2 d was only noted in the ZdTha group compared to the other 3 groups (*P* = 0.0240, 0.0020, and 0.0401 for control, Zd and Tha groups, respectively); this was followed by an increase to the level of pretreatment (Fig. 5B, Fig.6, Table S3).

### ZdTha Enhanced the Reduction in Tumor Vessel Permeability and Transient Vessel Normalization

**Figure 7:**
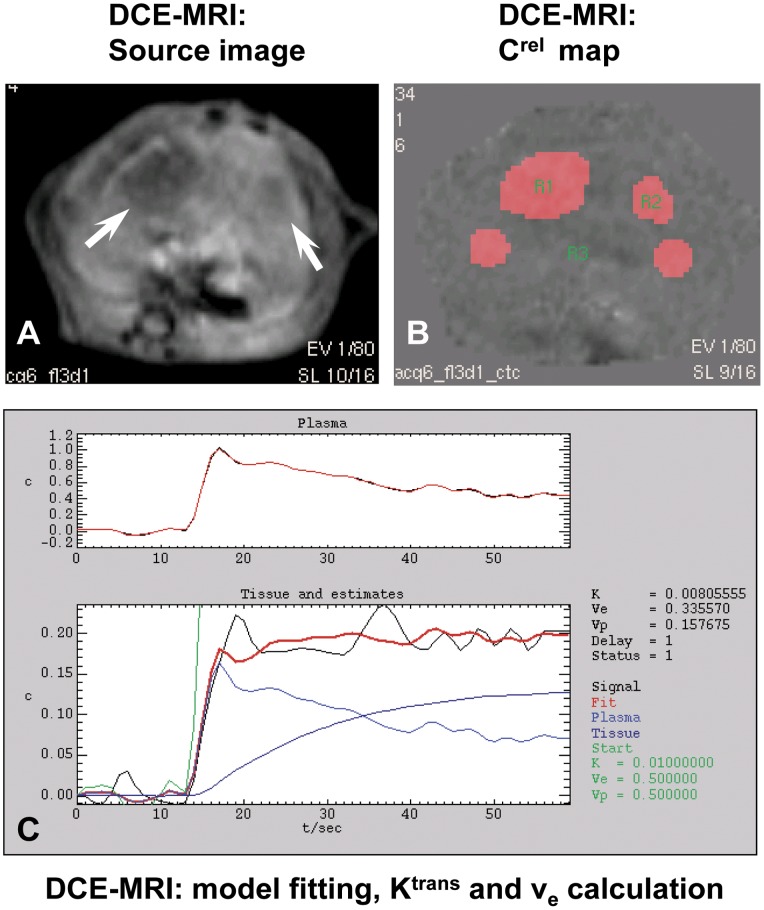
Representitive images illustrating the generation of K^trans^ and v_e_ from a case in the ZdTha group at day 2 after treatment. A: a source image from DCE-MRI showed two tumors (arrow) with reduced perfusion. **B:** a relative contrast agent concentration (C^rel^) map derived from a serial of source images of DCE-MRI. Four regions of interest (ROIs) were contoured on the source images: the two tumors (R1 and R2), the normal liver (R3), and the abdominal aorta (R4, not shown). Then the four ROIs were transferred onto the C^rel^ map. R4 was used to determine the arterial input function. **C:** The curves for the four ROIs were measured and fit with the Tofts and Kermode model to calculate the K^trans^ and v_e_. The optimal overlap of the black line (signal) on the red line (fit) indicated the best fitting of tumor signal curve with the model curve.

In both the Tha and control groups, K^trans^ showed a similar pattern of fluctuation over time as the tumor gradually matured. This suggested that the use of Tha alone did not induce definite tumor vessel permeability changes. Compared to the controls, the Zd and ZdTha groups showed significant decreases in K^trans^ (*P = *0.0010 and *P*<0.0001, respectively) at 4 h. This was probably the result of tumor vessel blockage and blood congestion induced by Zd. In the Zd group, this K^trans^ reduction was followed by a rebound, which stabilized at a level above the pretreatment level at 12 d. However, the K^trans^ of the ZdTha group remained consistently lower than that of controls at 2 d (*P = *0.0026) and 6 d (*P = *0.0500); it finally returned to the level of the controls at 12 d (*P = *0.7749). This indicated that the combined therapy of ZdTha provided an enhanced transient reduction of tumor vessel permeability (Fig. 5C, Fig.7, Table S4).

Similar to the changes observed in K^trans^, v_e_ decreased significantly in Zd (*P = *0.0407) and ZdTha (*P = *0.0459) groups compared to controls at 4 h. In the Zd group, this was followed by a return to the level of the controls from 2 d onwards. Contrarily, in the ZdTha group, the v_e_ decrease was significantly stronger than in the controls at 2 d (*P = *0.0420) and 12 d (*P = *0.0812), and was only at the level of the controls at 6 d. In the Tha group, the change in v_e_ was almost the same as that observed in the controls, except at 12 d, where v_e_ sharply rose; this indicated a relatively large amount of necrosis, which may explain the increase in extracellular fluid in that group compared to the others (Fig. 5D, Fig.7, Table S4).

### Multiple Linear Regression Analysis

A linear regression analysis was performed to assess the strength of the relationships between the MRI-derived parameters and the tumor volume changes after treatment. The analysis was performed only for the combined therapy, because the ZdTha treatment showed the best antitumor efficacy among all treatments. The results showed that the ADC change was significantly negatively correlated with the tumor volume change at 12 d compared to pretreatment values. A stepwise multiple linear regression analysis further demonstrated that the ADC change at 12 d was the only independent predictor of tumor volume changes (r = -0.652, *P = *0.030).

### Changes in Circulating Biomarkers and Microvessels after Treatment

The EPC levels were not significantly different between pre- and post Zd treatment samples. There was only a significant increase in plasma SDF-1α at 2 d after Zd administration compared to pretreatment (*P = *0.0136). Circulating EPCs and SDF-1α were higher at 4 h compared to pretreatment, but the differences were not significant (Table S5). The CD105^+^ MVD was not significantly different among the four treatment groups (Fig. S2).

## Discussion

The present study demonstrated that a single dose of Zd caused rapid vascular shutdown at 4 h, followed by tumor necrosis at 2 d, which delayed tumor growth compared to control tumors. However, tumor growth was not completely inhibited, and tumors began to relapse after 2 d, despite a massive central necrotic area in the tumor induced by the agent. These results were consistent with previous findings [26], and were supported with the multiparametric MRI techniques applied in this study. The multiparametric MRIs include T2WI and CE-T1WI for tumor morphologic measurement, DWI for cell density evaluation and for differentiating viable tumors (low ADC) from necrotic tumors (high ADC) [27], and DCE- or DSC-MRI derived parameters for blood flow and permeability (rBV, rBF, K^trans^, v_e_) information. The K^trans^ was measured as the diffusive transport of low-molecular weight Gd chelate across the capillary endothelium.

Two factors may be associated with the failure of Zd therapy. One was that viable tumor cells remained in the peripheral rim, based on CE-T1WI observations. These viable cells obtain nutrition from neighboring normal liver tissues and vessels, which would then lead to rapid repopulation of tumor cells within the necrotic area [28]; the other factor, as suggested in the literature [29], was that the VDA could have enhanced the hypoxia in the microenvironment. This could have upregulated the expression of hypoxia inducible factor 1α (HIF-1α), stimulated the expression of angiogenic genes, and increased the level of circulating proangiogenic cytokines, like vascular endothelial growth factor and SDF-1α. In turn, those cytokines could mobilize and attract bone marrow–derived circulating EPCs to tumor vessels [18], [24]. VDA treatment may have induced either or both factors, and promoted tumor angiogenesis. In our study, tumors recurred in the Zd group, evidenced by the rapid restoration of rBV and rBF (sometimes exceeding pretreatment levels), increases in K^trans^ and v_e_, and the gradual reduction of ADC that began at 2 d after treatment. The levels of plasma SDF-1α and circulating EPCs were higher at 4 h compared to pretreatment, but the differences were not significant. Therefore, our results did not show a conclusive association between tumor neovascularization processes and the mobilization of EPCs induced by Zd.

Tha is a derivative of glutamic acid; it was used as a sedative in the 1950s. Later, it was withdrawn from the market, due to its severe teratogenic effects. It was reintroduced into clinical practice, when its antiangiogenic properties were discovered in a rabbit cornea model [30]. Currently, Tha is under evaluation in early-phase clinical trials for the treatment of various types of solid tumors [31]. In general, Tha effects can be attributed to two types of mechanisms. First, Tha indirectly inhibits angiogenesis via tumor necrosis factor and the prostaglandin E pathway [32]. It was shown that Tha could effectively inhibit tumor neoangiogenesis, but only in the early stages of tumor formation [7]; it had little or no effect on full-grown tumors. Our results were consistent with those findings; we treated tumors only after they had grown larger than 0.8 cm in diameter, and we found no significant tumor growth delay with Tha treatment alone compared to the control group. In a second mechanism, Tha can directly induce apoptosis or G1 growth arrest [33]. In our study, the whole tumor ADC in the Tha group decreased at 2 d. This was followed by a significant rise at 6 d compared to the controls. This suggested that Tha increased tumor cell apoptosis, which resulted in a reduction in cell density and an increase in water molecule diffusion. This was accompanied by an elevation in the extravascular space volume, indicated by v_e_, and a reduction in rBF at 12 d after Tha therapy. These direct effects of Tha were observed in our study and verified by TUNEL measurements of apoptosis.

Apparently, when used alone, neither of the two agents tested were fully effective treatments. However, this study demonstrated that the combination of Zd with Tha enhanced overall antitumor efficacy. This improved effect may arise from several mechanisms.

In one potential mechanism, Zd and Tha have synergistic effects on solid tumors. As mentioned before, a single dose of Tha did not reduce the size of full-grown tumors. However, we found that ZdTha significantly reduced tumor growth compared to other treatments. This might be explained by the Zd-induced tumor necrosis, which then promote tumor angiogenesis. In the combined approach, this Zd effect could provide the appropriate conditions for Tha to indirectly inhibit angiogenesis [32], because Tha is effective only in the early stages of tumor formation [7]. We used T2WI for tumor volume measurement. MRI measure showed that ZdTha induced a significant delay in tumor growth compared to control and Tha treatments from 2 d to 12 d after treatment. Furthermore, tumor volume was significantly smaller after ZdTha compared to that after Zd from 2 d to 6 d after treatment. In addition to delaying tumor growth, ZdTha induced tumor necrosis in an additive manner. For instance, tumors rebounded rapidly with Zd alone, but with ZdTha, tumor cells underwent increased apoptosis induced by Tha. This contributed to the total significant increase in necrosis quantified with ADC maps and finally verified with HE staining. Consequently, the combined ZdTha showed a significantly smaller viable tumor rim on the CE-T1WIs. This finding was further supported with a stepwise linear regression analysis, which showed that the change in ADC at 12 d compared to baseline was the only independent predictor of tumor volume change with the combined therapy. Because ADC was negatively correlated with tumor volume change over time, an increase in ADC (fewer constraints on mobility) would indicate less viable cells and more necrosis. Therefore, ADC represented a good imaging biomarker for evaluating tumor response after treatment [8], [9], [20], [34].

Another mechanism might be that Tha induced the rapid onset of functional tumor vessel normalization only when used in combination with Zd. In both preclinical and clinical studies, emerging evidence has supported the hypothesis that certain antiangiogenic agents transiently “normalized” the tumor vessel, which then improved oxygen and drug delivery [35]. In another report [36], Tha was found to induce vessel maturation by stimulating mural cell coverage; thus, Tha rescued vessel wall defects. The *in vivo* MRI methods [16] applied here demonstrated a significant reduction of K^trans^, i.e., a prolonged reduction of tumor vessel permeability. This was only found with ZdTha from 4 h to 6 d, but not with Zd or Tha alone. Also, the v_e_ decrease was transiently significantly stronger compared to controls from 4 h to 2 d, which could not be seen with Zd or Tha alone. A reduction of rBV from 4 h to 2 d and a transient increase in rBF after 2 d were also demonstrated with ZdTha. This was most likely due to the pruning and normalization of tumor vasculature.

The functional information obtained from MRI-derived parameters provided imaging evidence of vessel normalization induced by Tha [14]. The normalization window observed in our study was about 6 d, which was consistent with that reported in a previous study in mice [37]. Therefore, the effect of Zd may be maintained and enhanced due to improved blood flow induced by Tha, which improves oxygen and drug delivery during the normalization of tumor vessels [38].

There were some study limitations. First, imaging small animals with a clinical MR scanner was technically challenging, particularly for the liver, because abdominal respiratory movement in rats cannot be completely eliminated during MRI. Even when we implemented previously optimized MR sequences [15], movement continued to cause some variations in the MRI measurements of this study. Second, although relaxometry is a useful MRI technique for quantitative diagnosis at the lesion level, it was not used in the present study, due to the heavy work load and the relatively complicated post processing procedures. Third, instead of doing microvessel staining at each time point, we only performed it at the end (day 12) of the experiment. Therefore, further experiments with immunohistochemical staining at more time points are warranted to better define the therapeutic effects of different agents.

In summary, our randomized, controlled study demonstrated that the ZdTha combination enhanced overall antitumor efficacy due to synergistic effects. ZdTha induced cumulative tumor necrosis, and thus, significantly delayed tumor volume growth and reduced the viable tumor rim; ZdTha also reduced tumor vessel permeability and improved tumor hemodynamic indexes via a transient normalization of tumor vasculature induced by Tha. Finally, the ADC value at 12 d was shown to be an independent predictor of changes in tumor volume. These therapeutic effects were successfully tracked and evaluated with *in vivo* multiparametric MRI.

## Supporting Information

Figure S1 **Tumor necrosis and viable rim seen on MRI.** Among the four treatments, ZdTha was most effective at causing tumor necrosis and reducing the viable tumor rim. (**A**) Representative axial images of liver tumors in the right (R) and left (L) lobes. Images are contrast-enhanced T1-weighted images (CE-T1WIs; TR/TE = 535/9.2 ms). **Top row:** A significantly smaller tumor rim diameter (yellow arrow) and larger necrosis (n) were detected in the ZdTha compared to the other groups after contrast agent injection. This was verified on macroscopic images of HE stained slices. **Row 2:** The right tumor regrew rapidly from 2 d after Zd treatment with reduced central necrosis (n) and a very thick tumor rim (yellow arrow); confirmed with macroscopic findings (HE image); **Row 3:** The right tumor grew remarkably during Tha treatment with necrotic areas (n) distributed irregularly and inhomogeneously within the viable tumor (white arrow). This was consistent with the macroscopic findings (HE image); **Bottom** **row:** The right tumor grew significantly during the 12-d experiment, with only a few small foci of spontaneous necrosis (n) within the viable tumor in the control group. (**B**) Tumor rim diameter changes were compared between Zd and ZdTha treatments, measured on CE-T1WIs. The mean relative diameter of the tumor rims in the ZdTha group was significantly smaller than that in the Zd group at 6 d and 12 d after treatment (*P = *0.0427 and *P = *0.0050, respectively). (**C**) Tumor necrosis volume changes were compared between Zd and ZdTha treatments, measured on the CE-T1WIs. The mean relative volume of necrosis visualized in the ZdTha group was significantly larger compared to that of the Zd group at 6 d and 12 d after treatment (*P = *0.0026 and *P = *0.0140, respectively).
(TIF)

Figure S2 **Comparison of microvessel density (MVD) on immunohistochemically stained slices.** (**A**) **Top row:** Whole tumor sections were scanned to identify areas with MVD at low magnification (50×). **Bottom row:** Microvessels detected with CD105 fluorescent antibody binding were counted per field at high magnification (400×). (**B**) No significant difference between groups was found in the CD105-positive MVDs.
(TIF)

Table S1 **Changes in tumor volume, from pretreatment to different times after treatment, based on T2-weighted images (T2WI).**
(DOC)

Table S2 **Changes in tumor relative apparent diffusion coefficient (rADC), from pretreatment values to different time points after treatment.**
(DOC)

Table S3 **Changes in tumor functional parameters compared to pretreatment values, derived from dynamic susceptibility contrast-enhanced magnetic resonance imaging.**
(DOC)

Table S4 **Changes in tumor functional parameters compared to pretreatment values, derived from dynamic contrast-enhanced magnetic resonance imaging.**
(DOC)

Table S5 **Changes in circulating endothelial progenitor cells (EPCs) and plasma stromal cell-derived factor-1α (SDF-1α) in both Zd and ZdTha groups.**
(DOC)

Text S1 **MRI Analysis.**
(DOC)
