# Supplementary figures and images for: Enhanced Antitumor Efficacy of a Vascular Disrupting Agent Combined with an Antiangiogenic in a Rat Liver Tumor Model Evaluated by Multiparametric MRI
Source: PLoS One. 2012 Jul 18;7(7):e41140. doi: 10.1371/journal.pone.0041140 (PMC3399789; doi:10.1371/journal.pone.0041140)

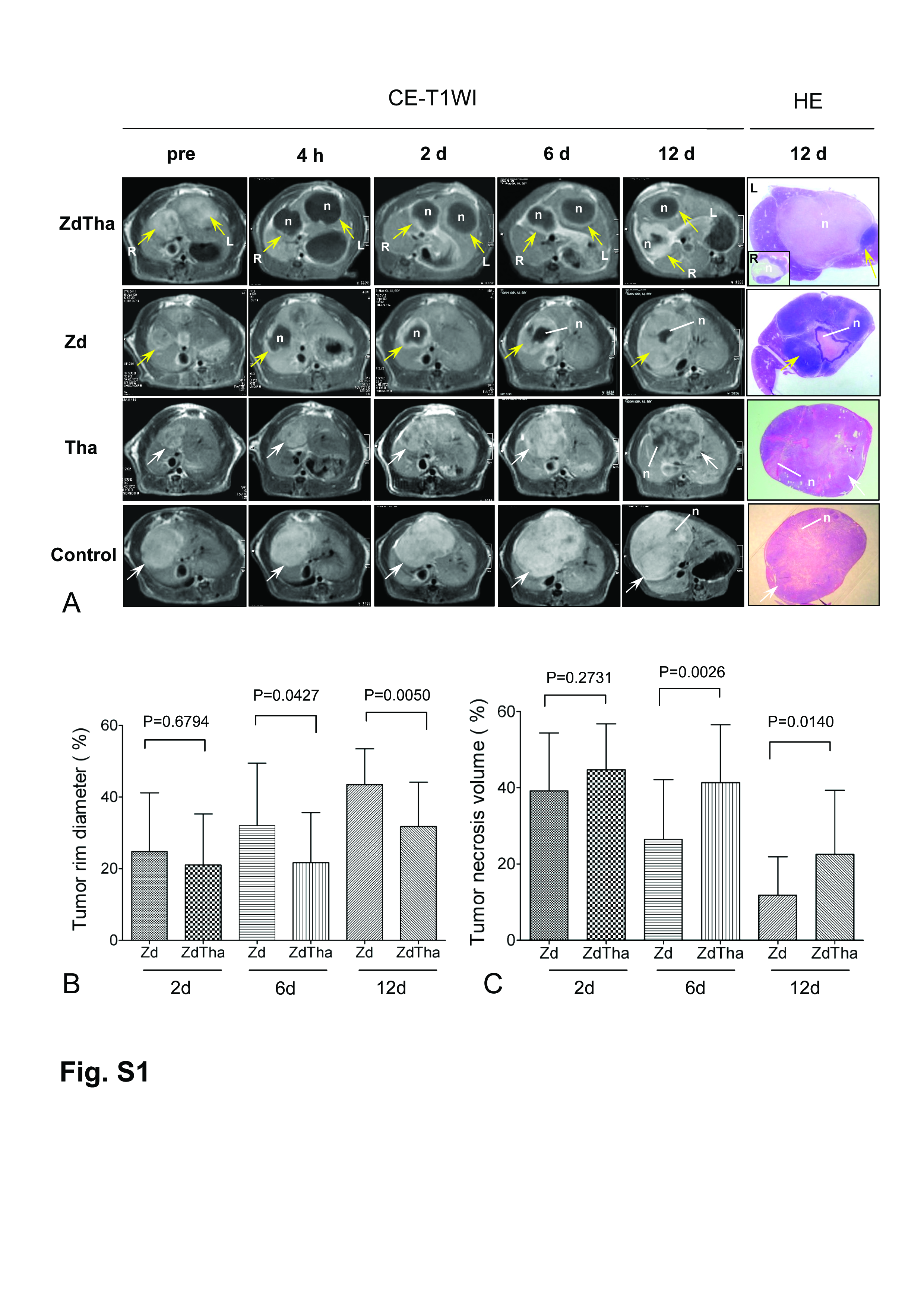

Supplement: Figure S1 — Tumor necrosis and viable rim seen on MRI. Among the four treatments, ZdTha was most effective at causing tumor necrosis and reducing the viable tumor rim. (A) Representative axial images of liver tumors in the right (R) and left (L) lobes. Images are contrast-enhanced T1-weighted images (CE-T1WIs; TR/TE = 535/9.2 ms). Top row: A significantly smaller tumor rim diameter (yellow arrow) and larger necrosis (n) were detected in the ZdTha compared to the other groups after contrast agent injection. This was verified on macroscopic images of HE stained slices. Row 2: The right tumor regrew rapidly from 2 d after Zd treatment with reduced central necrosis (n) and a very thick tumor rim (yellow arrow); confirmed with macroscopic findings (HE image); Row 3: The right tumor grew remarkably during Tha treatment with necrotic areas (n) distributed irregularly and inhomogeneously within the viable tumor (white arrow). This was consistent with the macroscopic findings (HE image); Bottom row: The right tumor grew significantly during the 12-d experiment, with only a few small foci of spontaneous necrosis (n) within the viable tumor in the control group. (B) Tumor rim diameter changes were compared between Zd and ZdTha treatments, measured on CE-T1WIs. The mean relative diameter of the tumor rims in the ZdTha group was significantly smaller than that in the Zd group at 6 d and 12 d after treatment (P = 0.0427 and P = 0.0050, respectively). (C) Tumor necrosis volume changes were compared between Zd and ZdTha treatments, measured on the CE-T1WIs. The mean relative volume of necrosis visualized in the ZdTha group was significantly larger compared to that of the Zd group at 6 d and 12 d after treatment (P = 0.0026 and P = 0.0140, respectively). (TIF) [file pone.0041140.s001.tif]

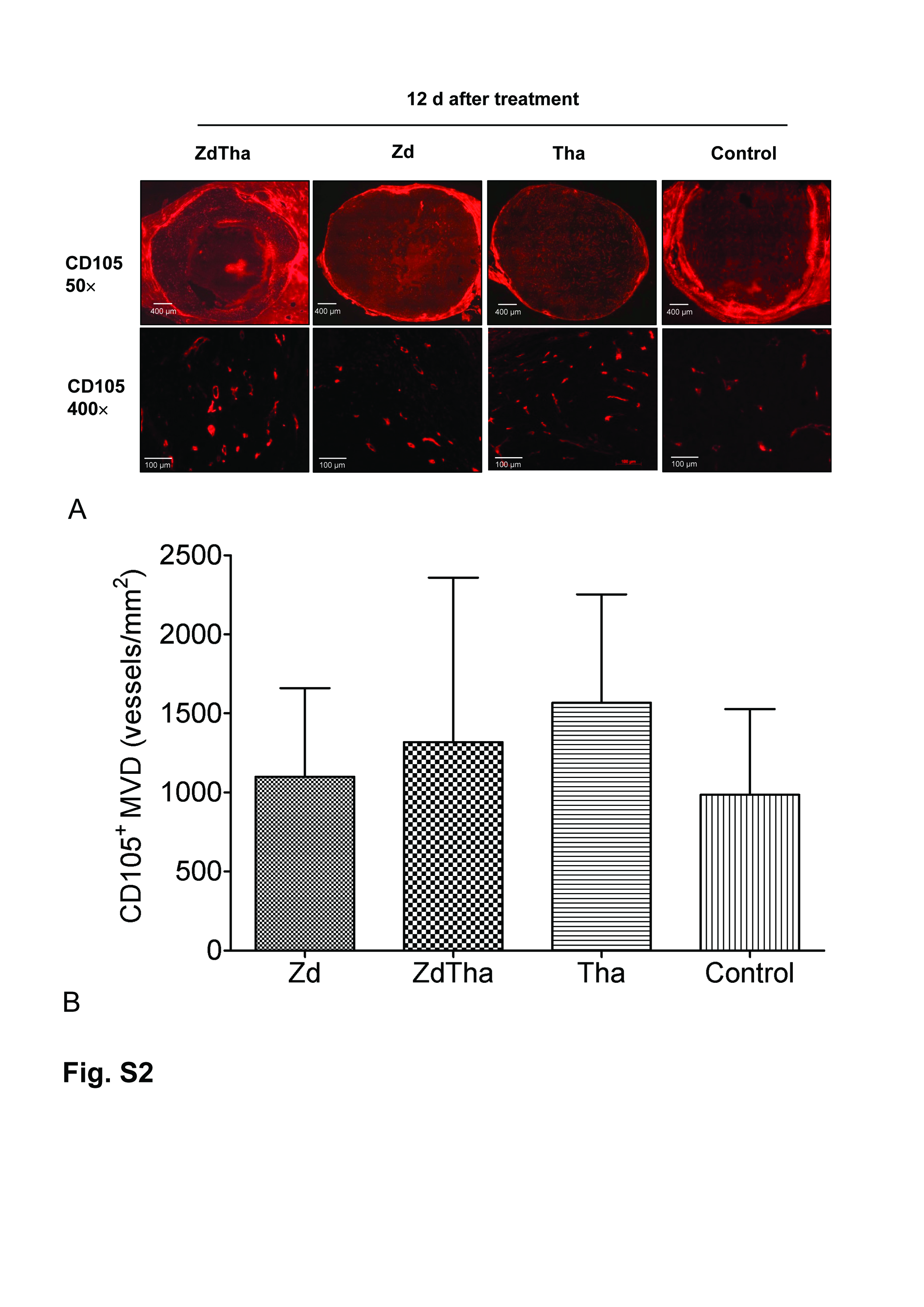

Supplement: Figure S2 — Comparison of microvessel density (MVD) on immunohistochemically stained slices. (A) Top row: Whole tumor sections were scanned to identify areas with MVD at low magnification (50×). Bottom row: Microvessels detected with CD105 fluorescent antibody binding were counted per field at high magnification (400×). (B) No significant difference between groups was found in the CD105-positive MVDs. (TIF) [file pone.0041140.s002.tif]
