# Supplementary material for: Enhanced Antitumor Efficacy of a Vascular Disrupting Agent Combined with an Antiangiogenic in a Rat Liver Tumor Model Evaluated by Multiparametric MRI
Source: PLoS One. 2012 Jul 18;7(7):e41140. doi: 10.1371/journal.pone.0041140 (PMC3399789; doi:10.1371/journal.pone.0041140)
Supplement: Table S1 — Changes in tumor volume, from pretreatment to different times after treatment, based on T2-weighted images (T2WI). (DOC) [file pone.0041140.s003.doc]

**Table S1. Changes in tumor volume, from pretreatment to different times after treatment, based on T2-weighted images (T2WI)**

| **Treatment groups** | **4 h** | **2 d** | **6 d** | **12 d** |
| --- | --- | --- | --- | --- |
|  | **Tumor volume change on T2WI** (%) | | | |
| **Zd** | 41.9 ± 19.3 | 60.9 ± 33.3 | 123.1 ± 118.6 | 477.3 ± 151.1 |
| **ZdTha** | 22.7 ± 14.8 | 15.2 ± 7.0 | 59.9 ± 16.0 | 213.5 ± 243.8 |
| **Tha** | 19.0 ± 12.8 | 72.5 ± 28.1 | 282.5 ± 146.9 | 982.8 ± 216.2 |
| **Control** | 33.0 ± 4.0 | 133.0 ± 60.4 | 460.2 ± 288.3 | 1418.9 ± 419.1 |
| **P values** |  |  |  |  |
| Ctrl vs. Zd | 1.0000 | <0.0001 | <0.0001 | 0.0004 |
| Ctrl vs. ZdTha | 0.3228 | <0.0001 | <0.0001 | <0.0004 |
| Ctrl vs. Tha | 0.0511 | 0.0007 | 0.0529 | 0.7090 |
| Zd vs. ZdTha | 0.4934 | <0.0001 | 0.0372 | 0.0629 |
| Zd vs. Tha | 0.0565 | 0.1285 | <0.0001 | 0.0007 |
| ZdTha vs. Tha | 1.0000 | <0.0001 | <0.0001 | <0.0001 |

Note: Data represents the mean ± SD; Zd = zd6126; ZdTha = zd6126 + thalidomide; Tha = thalidomide; Ctrl = control.
