# Supplementary material for: Enhanced Antitumor Efficacy of a Vascular Disrupting Agent Combined with an Antiangiogenic in a Rat Liver Tumor Model Evaluated by Multiparametric MRI
Source: PLoS One. 2012 Jul 18;7(7):e41140. doi: 10.1371/journal.pone.0041140 (PMC3399789; doi:10.1371/journal.pone.0041140)
Supplement: Table S2 — Changes in tumor relative apparent diffusion coefficient (rADC), from pretreatment values to different time points after treatment. (DOC) [file pone.0041140.s004.doc]

**Table S2. Changes in tumor relative apparent diffusion coefficient (rADC), from pretreatment values to different time points after treatment**

| **Treatment groups** | **4 h** | **2 d** | **6 d** | **12 d** |
| --- | --- | --- | --- | --- |
|  | **Tumor rADC change compared to baseline** (%) | | | |
| **Zd** | -28.3 ± 17.5 | 28.4 ± 17.9 | 29.4 ± 29.0 | 0.9 ± 22.7 |
| **ZdTha** | -20.9 ± 15.0 | 58.7 ± 47.4 | 75.8 ± 49.3 | 35.9 ± 63.9 |
| **Tha** | 1.6 ± 25.7 | -11.5 ± 14.1 | 33.0 ± 34.0 | 28.8 ± 36.5 |
| **Control** | 5.1 ± 23.9 | 14.9 ± 17.4 | 7.1 ± 5.8 | -2.6 ± 7.8 |
| **P values** |  |  |  |  |
| Ctrl vs. Zd | <0.0001 | 0.0751 | 0.1229 | 0.6295 |
| Ctrl vs. ZdTha | <0.0001 | 0.0003 | 0.0386 | 0.0190 |
| Ctrl vs. Tha | 0.1810 | 0.0003 | 0.0858 | 0.0160 |
| Zd vs. ZdTha | 0.0722 | 0.0256 | 0.5051 | 0.0251 |
| Zd vs. Tha | <0.0001 | <0.0001 | 0.8919 | 0.0026 |
| ZdTha vs. Tha | 0.0012 | <0.0001 | 0.5688 | 0.3975 |

Note: Data represents the mean ± SD; Zd = zd6126; ZdTha = zd6126 + thalidomide; Tha = thalidomide; Ctrl = control.
