# Supplementary material for: Enhanced Antitumor Efficacy of a Vascular Disrupting Agent Combined with an Antiangiogenic in a Rat Liver Tumor Model Evaluated by Multiparametric MRI
Source: PLoS One. 2012 Jul 18;7(7):e41140. doi: 10.1371/journal.pone.0041140 (PMC3399789; doi:10.1371/journal.pone.0041140)
Supplement: Table S3 — Changes in tumor functional parameters compared to pretreatment values, derived from dynamic susceptibility contrast-enhanced magnetic resonance imaging. (DOC) [file pone.0041140.s005.doc]

**Table S3*.* Changes in tumor functional parameters compared to pretreatment values, derived from dynamic susceptibility contrast-enhanced magnetic resonance imaging**

| **Treatment groups** | **4 h** | **2 d** | **6 d** | **12 d** |
| --- | --- | --- | --- | --- |
|  | **Tumor rBV change** (%) | | | |
| **Zd** | -59.4 ± 30.0 | 7.5 ± 38.0 | 16.7 ± 39.0 | 9.3 ± 34.6 |
| **ZdTha** | -79.3 ± 16.2 | -66.3 ± 29.9 | -54.2 ± 32.7 | -38.0 ± 12 |
| **Tha** | -13.3 ±43.8 | -40.7 ± 28.1 | -42.7 ±45.2 | -69.4 ±35.7 |
| **Control** | -9.6 ± 15 | -27.0 ± 22.4 | -47.1 ± 8.4 | -62 ± 8.9 |
| **P values** |  |  |  |  |
| ZdTha vs. Ctrl | <0.0001 | 0.0020 | 0.2578 | 0.0832 |
| Zd vs. Ctrl | <0.0001 | 0.4003 | 0.0142 | <0.0001 |
| Tha vs. Ctrl | 0.1698 | 0.0683 | 0.6216 | 0.0759 |
| ZdTha vs. Zd | 0.0304 | <0.0001 | 0.0003 | 0.0979 |
| ZdTha vs. Tha | <0.0001 | 0.0649 | 0.4283 | 0.0031 |
| Zd vs. Tha | <0.0001 | 0.0042 | 0.0011 | <0.0001 |
|  | **Tumor rBF change** (%) | | | |
| **Zd** | -8.2 ± 8.5 | 6.4 ± 4.2 | 0.7 ± 6.4 | 5.6 ± 14.0 |
| **ZdTha** | -13.7 ± 5.3 | -34.2 ± 14.0 | -1.1 ± 8.4 | -2.2 ± 8.0 |
| **Tha** | 3.4 ± 5.6 | 1.1 ± 5.9 | 5.6 ± 7.5 | -7.2 ± 9.8 |
| **Control** | 2.0 ± 4.4 | 1.0 ± 4.0 | 4.2 ± 3.8 | -3.8 ± 4.2 |
| **P value** |  |  |  |  |
| ZdTha vs. Ctrl | 0.1086 | 0.0240 | 0.6400 | 0.7062 |
| Zd vs. Ctrl | 0.4653 | 0.7502 | 0.2664 | 0.3150 |
| Tha vs. Ctrl | 0.9340 | 0.7139 | 0.3262 | 0.6809 |
| ZdTha vs. Zd | 0.3733 | 0.0020 | 0.4137 | 0.1478 |
| ZdTha vs. Tha | 0.0368 | 0.0401 | 0.3579 | 0.9651 |
| Zd vs. Tha | 0.3346 | 0.0858 | 0.9822 | 0.1439 |

Note: Data represent the mean ± SD; rBV = relative blood volume; rBF = relative blood flow; Zd = zd6126; ZdTha = zd6126 + thalidomide; Tha = thalidomide; Ctrl = control.
