# Supplementary material for: Enhanced Antitumor Efficacy of a Vascular Disrupting Agent Combined with an Antiangiogenic in a Rat Liver Tumor Model Evaluated by Multiparametric MRI
Source: PLoS One. 2012 Jul 18;7(7):e41140. doi: 10.1371/journal.pone.0041140 (PMC3399789; doi:10.1371/journal.pone.0041140)
Supplement: Table S4 — Changes in tumor functional parameters compared to pretreatment values, derived from dynamic contrast-enhanced magnetic resonance imaging. (DOC) [file pone.0041140.s006.doc]

**Table S4. Changes in tumor functional parameters compared to pretreatment values, derived from dynamic contrast-enhanced magnetic resonance imaging**

| **Treatment groups** | **4 h** | **2 d** | **6 d** | **12 d** |
| --- | --- | --- | --- | --- |
|  | **Tumor K^trans^ change** (%) | | | |
| **Zd** | -60.5 ± 15.2 | -20.8 ± 54.6 | 45.1 ± 37.2 | 31.2 ± 41.6 |
| **ZdTha** | -85.0 ± 22.2 | -70.4 ± 39.4 | -64.1 ± 40.4 | -15.3 ± 50.1 |
| **Tha** | -38.3 ± 27.6 | 40 ± 26.9 | 19.9 ± 38.1 | 27.2 ± 74.4 |
| **Control** | -37.4 ± 59.7 | 7.8 ± 38.4 | 1.5 ± 54.8 | -35.4 ± 21.4 |
| ***P values*** |  |  |  |  |
| ZdTha vs. Ctrl | <0.0001 | 0.0026 | 0.0500 | 0.7749 |
| Zd vs. Ctrl | 0.0010 | 0.0509 | 0.6695 | 0.3712 |
| Tha vs. Ctrl | 0.7170 | 0.8331 | 0.7347 | 0.6969 |
| ZdTha vs. Zd | 0.2822 | 0.2092 | 0.0154 | 0.5456 |
| ZdTha vs. Tha | <0.0001 | 0.0003 | 0.0092 | 0.9232 |
| Zd vs. Tha | 0.0004 | 0.0126 | 0.8833 | 0.6014 |
|  | **Tumor v_e_ change** (%) | | | |
| **Zd** | -60.5 ± 22.9 | 41.6 ± 52.3 | 33.0 ± 38.7 | 10.7 ± 27.4 |
| **ZdTha** | -40.5 ± 26.1 | -41.8 ± 33.1 | 7.4 ± 50 | -33.0 ± 30.5 |
| **Tha** | 40.7 ± 32.4 | 32.8 ± 16.0 | 33.3 ± 23.2 | 111.6 ± 38.8 |
| **Control** | -7.1 ± 19.2 | -10.7 ± 10.4 | -14.5 ± 18.1 | 12.6 ± 28.5 |
| ***P values*** |  |  |  |  |
| ZdTha vs. Ctrl | 0.0459 | 0.0420 | 0.9763 | 0.0812 |
| Zd vs. Ctrl | 0.0407 | 0.7178 | 0.5058 | 0.6347 |
| Tha vs. Ctrl | 0.7482 | 0.1408 | 0.1818 | 0.0636 |
| ZdTha vs. Zd | 0.7501 | 0.0885 | 0.5354 | 0.1875 |
| ZdTha vs. Tha | 0.0102 | 0.0057 | 0.2087 | 0.0010 |
| Zd vs. Tha | 0.0061 | 0.2860 | 0.5862 | 0.0222 |

Note: Data represent the mean ± SD. K^trans^ = vascular permeability coefficient; v_e_ = extravascular volume; Zd = zd6126; ZdTha = zd6126 + thalidomide; Tha = thalidomide; Ctrl = control.
