# Supplementary material for: Enhanced Antitumor Efficacy of a Vascular Disrupting Agent Combined with an Antiangiogenic in a Rat Liver Tumor Model Evaluated by Multiparametric MRI
Source: PLoS One. 2012 Jul 18;7(7):e41140. doi: 10.1371/journal.pone.0041140 (PMC3399789; doi:10.1371/journal.pone.0041140)
Supplement: Table S5 — Changes in circulating endothelial progenitor cells (EPCs) and plasma stromal cell-derived factor-1α (SDF-1α) in both Zd and ZdTha groups. (DOC) [file pone.0041140.s007.doc]

**Table S5. Changes in circulating endothelial progenitor cells (EPCs) and plasma stromal cell-derived factor-1α (SDF-1α) in both Zd and ZdTha groups**

| **Treatment groups** | | **pre** | **4h** | **2d** | ***P* value** | | |
| --- | --- | --- | --- | --- | --- | --- | --- |
|  |  | **Circulating EPCs** (%)  **(CD105^+^ flk-1^+^)** | | | pre vs. 4h | | pre vs. 2d |
| **Zd** | | 1.03 ± 1.32 | 3.42 ± 5.50 | 1.34 ± 1.06 | 0.3704 | | 0.3996 |
| **ZdTha** | | 0.57 ± 0.14 | 1.22 ± 1.74 | 0.72 ± 0.23 | 0.5126 | | 0.2655 |
| ***P* value** | | 0.8696 | 0.1812 | 0.1826 |  | |  |
| Zd vs. ZdTha | |  |  |  |  | |  |
| **Plasma SDF-1α** (pg/ml) | | | | | | | |
| **Zd** | 0.82 ± 0.33 | | 0.97 ± 0.31 | 0.65 ± 0.26 | 0.1913 | 0.0136 | |
| **ZdTha** | 0.49 ± 0.22 | | 0.58 ± 0.28 | 0.52 ± 0.31 | 0.5795 | 0.6256 | |
| ***P* value** | 0.0636 | | 0.0699 | 0.4774 |  |  | |
| Zd vs. ZdTha |  |  |  |  |  |  | |

Note: Data represents the mean ± SD. Zd = zd6126; ZdTha = zd6126 + thalidomide.
