# Supplementary material for: Enhanced Antitumor Efficacy of a Vascular Disrupting Agent Combined with an Antiangiogenic in a Rat Liver Tumor Model Evaluated by Multiparametric MRI
Source: PLoS One. 2012 Jul 18;7(7):e41140. doi: 10.1371/journal.pone.0041140 (PMC3399789; doi:10.1371/journal.pone.0041140)
Supplement: Text S1 — MRI Analysis. (DOC) [file pone.0041140.s008.doc]

**SUPPORTING INFORMATION**

***Text S1: MRI Analysis***

*DWI*

For each lesion, freehand delineations were performed on all slices of the original DW images acquired with a *b*-value of 1000 s/mm^2^. These delineations were combined to form one three-dimensional volume of interest per lesion. The volume of interest was then automatically copied to all other *b*-value images and the average signal intensity per *b*-value was determined. Afterwards, the apparent diffusion coefficient (ADC) was calculated based on the common monoexponential diffusion model. To that end, a least squares solution of the following system of equations was solved:

$$S_{i}=S_{0}*e^{-b_{i}*ADC}$$

where S_i_ was the signal intensity measured on the *i*^th^ *b*-value image, acquired with the *b*-value *b_i_*, and S_0_ was an estimate of the signal intensity for a *b*-value of 0 s/mm^2^. The dataset with 10 *b*-values also allowed biexponential fitting, but this was not included in the current manuscript.

*Dynamic contrast-enhanced MRI (DCE-MRI)*

All DCE-MRI calculations were performed with BioMAP software. In the first step, pixel-wise, relative contrast agent concentration (C^rel^) maps were generated by a first order approximation of the signal changes:

$$C^{rel}(t)=\frac{S(t)-S_{b}}{S_{b}}$$

where S(t) was the measured signal at time point *t*, and S_b_ was the average signal intensity of the images before contrast administration. On the original DCE-MR images, three regions of interest (ROIs) were contoured: the whole tumor, the normal liver, and the abdominal aorta; the latter were used to determine the arterial input function (C_p_(t)). The ROIs were copied to the relative contrast agent concentration maps and the curves for these three tissues were measured. Next, the measured values were fit with the Tofts and Kermode model to calculate the permeability, K^trans^, as follows:

$$\frac{dC(t)}{dt}=K^{trans}(C_{p}(t)-\frac{C(t)}{v_{e}})$$

where C(t) was the tissue contrast, C_p_(t) the arterial input function, and v_e_ the extravascular volume (a value between 0 and 1).

*Dynamic susceptibility contrast-enhanced MRI (DSC-MRI)*

First, the contrast agent concentration (CTC) maps were calculated from the DSC-MRI data by solving the following equation for all pixels:

$$CTC(t)=-\frac{100}{TE}*\ln(\frac{S(t)}{S_{0}})$$

where S_0_ was the baseline signal intensity, TE was the echo time of the sequence, and S(t) was the measured signal intensity over time. Thereafter, delineations were performed on the lesions (CTC(t)) and arteries (AIF(t)), and the contrast agent concentrations for each were determined. The relative blood volume (rBV) was calculated by solving:

$$rBV=\frac{\kappa_{h}}{\rho}\frac{\int_{0}^{\infty} CTC(t)dt}{\int_{0}^{\infty} AIF(t)dt}$$

Finally, relative blood flow (rBF) values were calculated as rBF=rBV/MTT, where MTT was the mean transit time, calculated as:

$$MTT=\int_{t} R(t)dt$$

and the residual function, R(t), was calculated from

$$CTC(t)=\kappa*AIF(t)\otimes R(t)$$
